# Dr. Arthur C. Ford

**Published:** 1884-02

**Authors:** 


Arthur C. Ford, D. D. S., died at Palatka, Florida December 20th, 1883. Dr. Ford was a native of England, but resided in Georgia for many years, practicing dentistry in Atlanta, and at one time was President of the Georgia State Dental Society. For several years past, Dr. Ford has resided in Florida, when his failing health obliged him to leave Atlanta and seek a warmer climate. Dr. Ford was well known throughout the South as a skillful operator and a close student, manifesting great interest in his State and other Associations, and doing his utmost to elevate his profession. For the last two years Dr. Ford has been a member of the Corps of Clinical Operators of the Dental Department of the University of Maryland, and his loss thus early in life, will be deeply regretted by all who knew him.

